# Soft Modular Electronic Blocks (SMEBs): A Strategy for Tailored Wearable Health‐Monitoring Systems

**DOI:** 10.1002/advs.201801682

**Published:** 2018-12-20

**Authors:** Jaeyoung Yoon, Yunsik Joo, Eunho Oh, Byeongmoon Lee, Daesik Kim, Seunghwan Lee, Taehoon Kim, Junghwan Byun, Yongtaek Hong

**Affiliations:** ^1^ Department of Electrical and Computer Engineering Inter University Semiconductor Research Center (ISRC) Seoul National University Seoul 08826 Republic of Korea; ^2^ Department of Mechanical and Aerospace Engineering Institute of Advanced Machines and Design (IAMD) Soft Robotics Research Center (SRRC) Seoul National University Seoul 08826 Republic of Korea

**Keywords:** modular blocks, on‐skin assembly, stretchable hybrid electronics, stretchable platform, wearable healthcare devices

## Abstract

Precise monitoring of human body signals can be achieved by soft, conformal contact and precise arrangement of wearable devices to the desired body positions. So far, no design and fabrication methodology in soft wearable devices is able to address the variations in the form factor of the human body such as the various sizes and shapes of individual body parts, which can significantly cause misalignments and the corresponding inaccurate monitoring. Here, a concept of soft modular electronic blocks (SMEBs) enabling the assembly of soft wearable systems onto human skin with functions and layouts tailored to the form factors of individuals' bodies is presented. Three types of SMEBs are developed as fundamental building blocks for functional modularization. The physical design of SMEBs is optimized for a mechanically stable island‐bridge configuration. The prepared SMEBs can be integrated onto a target body part through rapid, room‐temperature (RT) assembly (<5 s) using an oxygen plasma‐induced siloxane bonding method. A soft metacarpophalangeal (MP) joints flexion monitoring system that is tailored to allow for accurate monitoring for multiple individuals with unique joint and hand sizes is demonstrated.

Recent advances in soft electronics have revolutionized noninvasive monitoring of body signals. Skin‐like conformability of this newer class of wearable devices has enabled an imperceptible mode of wearing and, at the same time, has improved the fidelity of data acquisition.^1^, ^2^, ^3^ In particular, the integration of advanced technologies, such as stretchable display^4^, ^5^ and therapy,^6^, ^7^ have achieved immediate feedback to users, in ways that were previously impossible. Representative applications include skin‐attachable soft sensors capable of acquiring body motion signals^1^, ^8^, ^9^ and/or vital signs (i.e., sweat,^10^, ^11^ electroencephalogram, electrocardiogram, and electromyogram^12^, ^13^, ^14^). More recent studies have focused on reducing the number of bulky wires, connected to the external electrical measurement equipment, to provide an optimized platform for ubiquitous systems. To this end, the concept of stretchable hybrid electronics (SHE) has entered into the mainstream, with a design that utilizes a slew of rigid circuit components (e.g., integrated circuit (IC) chips and passive elements).^15^, ^16^, ^17^, ^18^, ^19^ Methodologies of microfluidic assembly,^15^ self‐assembled 3D desgins,^16^ and fully printable assembly^17^, ^18^ showed various layouts and applications of SHE systems. In particular, the fragmented SHE design with minimized local hardness even validated the capability of in‐skin data processing without compromising skin‐conformability.^19^ Based on this trend, the potential of wearable devices has been further extended to allow for the refinement, computation, and/or wireless transmission of the acquired signals on the skin.^2^, ^15^, ^16^, ^19^, ^20^

Human bodies of different age and sex groups vary significantly in the size and the proportion of each part.^21^, ^22^, ^23^ Signal acquisition accuracy in soft wearable devices is severely vulnerable to this variation, because accurate monitoring of body signals heavily relies on the predefined position and arrangement of soft sensors that is difficult to change once designed. To alleviate this mispositioning issue, multichannel sensors in the form of arrays have been widely adopted.^24^ In this approach, multiple signals around the target body part were measured simultaneously via multichannel sensors to extract the best signal or to adjust the weight of the acquired signals.^25^, ^26^ However, this strategy not only required complex data processing or algorithm but also caused spatial inefficiency. Adjusting the position and arrangement of soft sensors in accordance with the form factor of individual user's body can be a promising alternative.^27^ In fact, the methods to modularize functional units in the form of rigid printed circuit boards or kits readily provided the effectiveness in tailoring the layout of wearable systems.^28^, ^29^ However, none of the existing fabrication methodologies and designs for wearable SHE system provide suitable platforms in this perspective.

Here, we present a concept of soft modular electronic blocks (SMEBs) that can be assembled directly onto human skin to construct the desired wearable system in a tailored layout for each user's body. The key challenges for the modularization of soft electronic system are mainly threefold: (1) functional modularization, (2) mechanical stability of the blocks, and (3) rapid, room‐temperature (RT) assembly directly onto the skin. From this perspective, we developed three types of SMEBs with distinct functions: sensor blocks (data acquisition), interconnect blocks (data transmission), and circuit blocks (data processing and display). Physical design of SMEBs was optimized for a mechanically stable island‐bridge configuration, and their mechanical stability was improved by introducing the strain‐relief layer into SMEBs. Rapid, on‐skin assembly of SMEBs was achieved by an RT siloxane bonding method (<5 s). To show the effectiveness of our concept, we demonstrated a soft metacarpophalangeal (MP) joints flexion monitoring system based on on‐skin assembly of SMEBs that allowed for accurate monitoring for multiple individuals with unique joint and hand sizes.

The form factors of human body (e.g., wrist circumference, leg‐to‐body ratio) vary too widely from person to person to use soft wearable devices with fixed designs. One of the most typical example is human hand. Recently, soft wearable glove‐based devices tracking hand motions and measuring grasp force are increasingly used for the diagnosis of hand‐injured patients and their hand rehabilitation.^30^, ^31^ Nonetheless, the difference in hand size is notable even out of the adult male group of the similar age (**Figure** **1**a–c). Specifically, the standard deviations of the breadth of the hand (Figure 1a), interval between two MP joints (Figure 1b) and length of middle finger (Figure 1c) are 3.7, 1.41, and 4.97 mm, respectively. This variation can cause critical misalignment and the corresponding inaccurate monitoring of hand signals. Provided that the wearable SHE system for the monitoring of MP joint flexions is designed according to the hand dimension of one specific user (S1), the use of the device may be inhibited in other users (S2, S3) due to the inaccurate signal acquisition (Figure 1d). Our approach is to modularize the wearable SHE system into fundamental unit blocks (SMEBs) with the ability to be assembled directly on human skin (Figure 1e). Three types of SMEBs (sensor, interconnect, and circuit blocks) were developed to provide a novel way to tailor the system layout for multiple individuals' body. This strategy is powerful because of (1) the integrated functionality (e.g., data analysis and display) based on SHE, (2) the ability to tailor the design for each user with adequate alignment, and (3) rapid, RT assembly.

**Figure 1:**
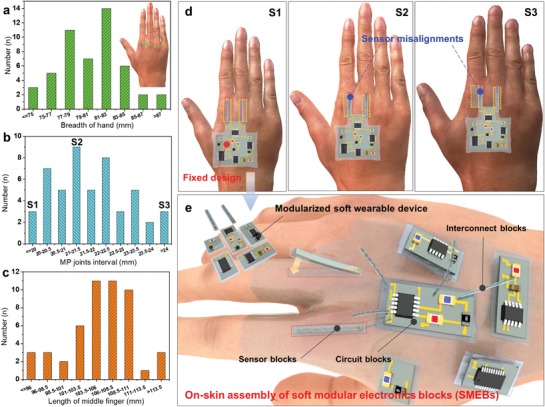
Modular stretchable hybrid electronics (SHE) for tailored wearable healthcare devices. Histogram of frequency distribution of a) the breadth of the hand, b) interval between two metacarpophalangeal (MP) joints, and c) length of middle finger in 50 adult male (20s and 30s) group. d) A soft wearable MP joints flexion sensing device designed for the subject S1 based on SHE. The device with a fixed design can cause misalignments and inaccurate monitoring when attached to the hands with different form factors (S2, S3). e) Schematic illustration of the modularized soft wearable device and its tailorable on‐skin assembly for the improved monitoring accuracy. The modular SHE can be realized by three types of soft modular electronic blocks (SMEBs).

The developed SMEBs have separate functionalities such as (i) high‐speed data processing, analysis and display (**Figure** **2**a), (ii) data acquisition (Figure 2b), and (iii) data transmission (Figure 2c). All blocks were fabricated by solution processes and their designs were optimized for a mechanically stable island‐bridge configuration (see the Experimental Section of the Supporting Information for the details on fabrication steps). The circuit block is a kind of hybrid‐type soft electronic circuits composed of IC chips, passive elements, and silver (Ag) electrodes (20–30 Ω cm^−1^ with 300 µm line width and 400 nm thickness) for high‐performance computational functionality (Figure 2a; Figure S1a, Supporting Information). The sensor block is a capacitive bending sensor for measuring joint flexions (Figure 2b; Figure S1b, Supporting Information).^32^ The interconnect block is a macroscale stretchable interconnect that bridges the circuit and sensor blocks in the form of the corrugated silver nanowires embedded in polydimethylsiloxane (termed corrugated AgNWs/PDMS) (Figure 2c; Figure S1c, Supporting Information). In particular, a polyethylene naphthalate (PEN) island and a strain‐relief layer (10:1 PDMS) is introduced for the robust strain isolation and modulus gradient effect, respectively.

**Figure 2:**
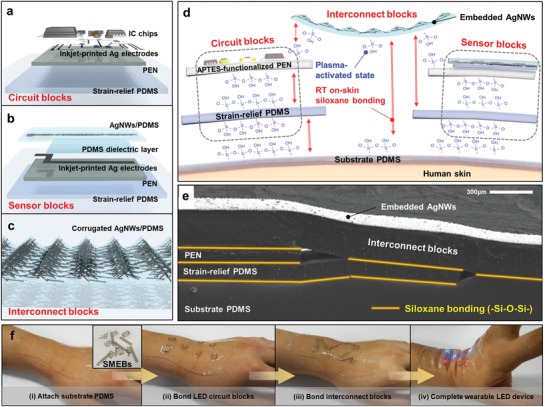
SMEBs and their assembly on human skin. a,b) Exploded view schematic illustrations of a) a circuit block and b) a sensor block (see Figure S1 of the Supporting Information for optical images). c) Section‐view schematic illustration of an interconnect block (see Figure S1 of the Supporting Information for a SEM image). d) Section‐view schematic illustration of on‐skin assembly of SMEBs and their bonding mechanism. e) SEM image of the assembled SMEBs through siloxane bonding (—Si—O—Si—). f) On‐skin SMEB assembly demonstration for a wearable LED device.

The assembly of these SMEBs was based on a siloxane bonding (—Si—O—Si—) method.^33^ Since the pristine PEN has no silanol (—Si—O—H—) groups on its surface, a (3‐aminopropyl)triethoxysilane treatment was carried out for ease of activation (Figure S2, Supporting Information). A 1 min 60W oxygen (O_2_) plasma treatment was performed to break the silanol groups in the surfaces of the SMEBs (Figure 2d). All the activated surfaces of the SMEBs formed irreversible bonding after direct physical contacts. Thus, the SMEBs could be readily assembled on a common substrate PDMS (Figure 2e). The bonding strength at the interfaces between the SMEBs ((i) PEN‐strain relief PDMS, (ii) strain‐relief PDMS‐substrate PDMS) was investigated by a 180° peeling test (Figure S3a, Supporting Information). The result showed that the peeling force of the interface (i) and (ii) was 0.2813 and 0.1415 N mm^−1^, respectively, both of which were stronger than that of the reference sample (Kapton‐PDMS). It is also assumed that the actual bonding strength was higher than the measured value, because it was impossible to delaminate the upper layers only from the lower layers and, instead, the bulk rupture of the lower layers occurred (Figure S3b, Supporting Information).

The plasma‐induced bonding method was able to perform at RT and could rapidly form the irreversible bonding (<5 s, see Video S1, Supporting Information) to the SMEBs of arbitrary shapes and sizes. A sufficiently strong siloxane bond could be formed even if a physical contact between the activated layers was exerted in around 30 min after the O_2_ plasma treatment, which is enough time to assemble SMEBs to complete a soft wearable device (Figure S3c, Supporting Information). To show the feasibility, we demonstrated a simple prototype of tailored wearable light‐emitting diode (LED) circuits (Figure 2f; Video S2, Supporting Information). Seven different circuit blocks with various shapes (triangles, rectangles, hexagons, and circles) and sizes and six interconnect blocks were assembled onto the skin within 3 min. The system could robustly operate without any electrical or mechanical failure even under the wrist deformations due to the robustness between the bonded layers and stretchable interconnect blocks.

The SMEB‐based tailored design in our approach was characterized by an island‐bridge structure. In this design, mechanical stability under stretching conditions generally relied on the strain‐free effect inside the island (PEN) and on the stability of the island boundaries in which a sharp modulus gradient could induce mechanical and/or electrical failure.^34^ We focused on the effect of the strain‐relief PDMS layer on the mechanical stability of the assembled SMEBs; In general, the larger the modulus mismatch between the layers, the higher the stress concentration occurred at the layer boundary. The introduction of the strain relief layer can reduce the stress peak at the boundary of each island structure by reducing abrupt modulus changes.^35^ The strain‐relief layer was designed with thickness of around 100 µm and about twofold area of the PEN. As the thickness of the strain‐relief layer decreases and the area increases, the functionalities of SMEBs could be maintained even at higher strains (Figure S4, Supporting Information); however, an excessively large area of the strain‐relief layer causes spatial inefficiency. A digital image correlation (DIC) method was used to experimentally obtain the exact strain distribution of the modular SHE system (four circuit blocks connected by four interconnect blocks in this case) (**Figure** **3**a). Three meaningful indices were defined to obtain a quantitative understanding of the mechanical stability of the assembled SMEBs: (i) ε_center_, the maximum principal strain at the center of a PEN island, (ii) AR_2%_, the area ratio of the strain‐free (<2%) domain to the whole PEN area,^15^ and (iii) ε_corner_, the strain at the corners of SMEBs. The DIC results show that ε_center_ of the SMEBs with and without the strain‐relief layer was equally ≈0%; however, the AR_2%_ value of the SMEB with the strain‐relief layer increased more than 15% compared to that without the strain‐relief layer (Figures S5a and S6, Supporting Information). This implies that the functionalities inside the circuit blocks or sensor blocks can be protected from the external strain more effectively. In terms of the boundary stability, the effect of the strain‐relief PDMS layer not only reduced ε_corner_ by around 10% at 50% uniaxial strain (Figure S5b, Supporting Information), but also guaranteed the gradual increase in strain near the SMEB boundary (Figure 3b). The strain at the interface of the PEN was reduced by approximately five times. These improvements in the boundary stability prevented the onset of rupture even at 50% uniaxial strain, at which the rupture occurred in the island without the strain‐relief PDMS (Figure S7, Supporting Information).

**Figure 3:**
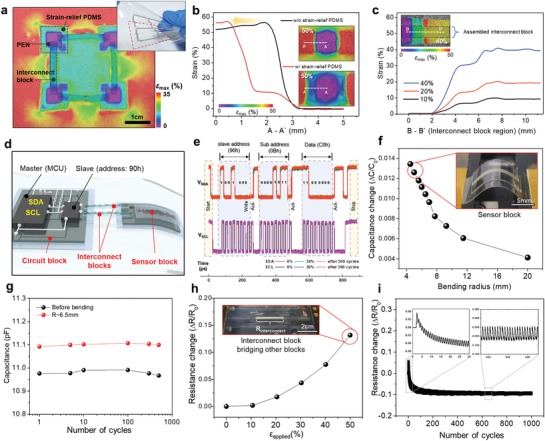
Mechanical and electrical stability of the assembled SMEBs. a) Large‐area surface strain mapping of the SMEBs under 30% biaxial strain. (Inset image: photograph of assembled SMEBs (9 circuit blocks and 12 interconnect blocks) under a stretching deformation.) b,c) Surface strain profiles of b) the circuit blocks without and with a strain‐relief PDMS layer (without interconnect block), and c) the interconnect blocks that bridge the neighboring circuit blocks. d) Schematic illustration of the assembled SHE system comprised of one circuit block, one sensor block, and two interconnect blocks connecting them. e) Reliability of I^2^C serial communication before and after 30% uniaxial stretching. Completely overlapped signals are shown after 1000 cycles of 30% uniaxial strain. f) Electrical characteristics of a bending sensor block under various bending conditions. g) Reliability of the bending sensor block during 500 bending cycles with a 6.5 mm bending radius. h) Electrical characteristic of an interconnect block as a function of uniaxial strain (0–50%). i) Reliability of the interconnect block during 1000 cycles of 25% uniaxial strain.

The stability of the inside and boundary of the islands also enables the robust connection between them through the interconnect blocks. AgNWs of the interconnect block formed an electrical contact with printed Ag pad via silver pastes. The surface strain near the contact region of the interconnect block showed a strain below 2% at a 40% strain, indicating the ability to maintain the stable electrical contact (Figure 3c). The surface strain profile of the interconnect block on the island showed more gradual increases than that of the island only. It was caused by the voids below the interconnect block where it was not bonded to other layers shown in Figure 2e. The strain at the interconnect block bonded onto the substrate PDMS showed the highest strain (>40%). This result indicated that the fragmented island design as well as the stretchable bridge design had a key role to reduce the discomfort of users

To analyze the electrical properties of the SMEBs, we composed a SHE system that can acquire and process the bending sensor signals. The system was designed to include the subcircuit of the demonstration to be presented hereafter, consisting of a circuit block, a bending sensor block, and two interconnect blocks (Figure 3d). The circuit block can be composed of high‐performance bulky IC chips (>7.8 mm) with more than ten leads, such as microcontroller unit (MCU) and analog‐to‐digital converter. The circuit block used in this work consists of two active elements—MCU and capacitance‐to‐digital converter (CDC)—and three passive elements—two resistor and one capacitor—as shown in schematic illustration of Figure 3d. To explore the electrical properties of the circuit block, we measured the voltage at the clock (SCL) and data (SDA) wire of I^2^C (interintegrated circuit) serial communication. On the basis of the mechanical stability as discussed above, the well‐engineered strain‐isolation region allowed that the high‐speed I^2^C communication signals (>100 kHz) can be transmitted stably even under 30% uniaxial strain. Even after stretching cycles to a uniaxial strain of 30%, the circuit block operated without any electrical degradations, showing almost overlapped *V*_SDA_ and *V*_SCL_ signals (Figure 3e).

In addition to circuit blocks, the sensor characteristics of bending sensor blocks were measured. The capacitance increased as the bending sensor block was bent as shown in Figure 3f. The blocks exhibited a capacitance change of 1.4% at a bending radius of about 5 mm. It is notable that the function of the sensor block was maintained without a significant performance change even after 500 bending cycles in Figure 3g. To investigate the electrical properties of the interconnect blocks connecting the circuit block and the sensor block, we prepared an interconnect block connected between two other blocks. The interconnect block shows a resistance change of around 14% at a uniaxial strain of 50% (Figure 3h); this change of resistance has negligible effect on the entire system. As described above, AgNWs were embedded in corrugated PDMS, and thus the resistance changes were lower than that of flat‐structured AgNWs/PDMS interconnect (Figure S8, Supporting Information). The flat‐structured AgNWs/PDMS showed resistance change of around 104% at a uniaxial strain of 50%. The interconnect block was also able to sustain its electrical connection over 1000 cycles to a uniaxial strain of 25% with a nearly constant resistance (Figure 3i). In the first stretch cycle, AgNWs cannot return to the initial alignment state, which results in a slight increase (≈1.5%) of resistance at 0% strain. In the next few cycles, the AgNWs begin to become realigned in the PDMS, with the result that the resistance at 0% strain tends to decrease slightly by around 1% every cycle.^36^ After that, the resistance at 0% strain saturates to the stable value (inset images of Figure 3i).

Rapid on‐skin implementation of integrated functionality and tailored design of wearable healthcare systems is essential for accurate monitoring and treatment for each user. The modular SHE concept presented here provides ideal platforms from this perspective. As a representative example, we demonstrated a tailored, wearable MP joints flexion monitoring system that could help the rehabilitation of the hand‐injured patients (**Figure** **4**). This system was composed of two bending sensor blocks, one signal‐processing circuit block, two display circuit blocks, and 14 interconnect blocks. According to the desired function and layout, the SMEBs can be substituted by other SMEBs with different functionalities, or additional SMEBs can easily be connected to them. All the SMEBs could be rapidly assembled onto the human hand in a tailored manner for any users of different ages regardless of their hand dimensions (Figure 4a,b). Furthermore, interconnect blocks bridging the fundamental SMEBs can be tailored in accordance with the layout of the entire system and assembled SMEBs (Figure S9, Supporting Information). In this system, the capacitance changes of the bending blocks according to the flexion of MP joints was converted into digital signal. The signal was updated every 20 ms in the CDC of the signal‐processing block. Then, the data were transmitted to the MCU through I^2^C serial communication. The MCU compared the data with reference data to display the flexion level through the display circuit blocks (see Figure S10 of the Supporting Information for the circuit diagram). The actual operation of the device that monitors the MP joints flexion is shown in Figure 4c and Video S3 (Supporting Information). When the power (3.3 V) is applied, the device sets the reference value at 0°, 45°, and 90° flexion through the calibration process (Figure S11, Supporting Information).

**Figure 4:**
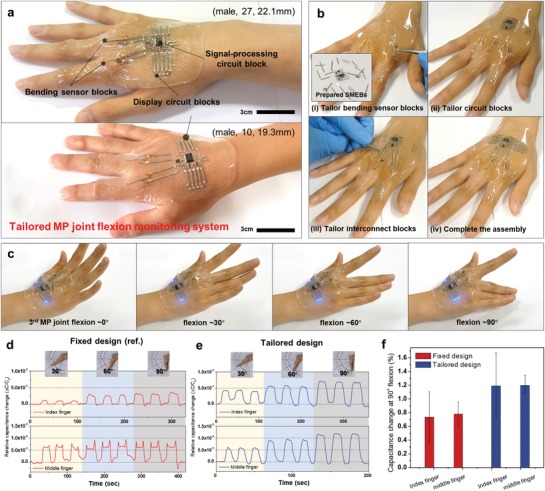
Tailored MP joints flexion monitoring system. a) Wearable MP joints flexion monitoring systems on the hand of an adult (top) and a child (bottom). The form factor of the individual subjects' hands is described in the following formats: (sex, age, and distance between 2nd and 3rd MP joints). b) The tailoring process flow of the system on a human hand. c) Sequential photographs of the completed system and its operation that monitors the flexion of the 3rd MP joint. d,e) Sequential monitoring of the hand flexion to 30°, 60°, 90° measured by the wearable device with d) a fixed design and e) a tailoring design. An identical subject (female, 26 year old, distance between 2nd and 3rd MP joints: 20.5 mm) performed this test. f) Accuracy of the sensors of the device with a fixed design and a tailoring design (five subjects).

The effectiveness of the SMEB‐based approach was validated by comparing the signal acquisition accuracy between the device with a tailored design and its counterpart with a fixed design. Figure 4d,e shows obtained signals for the five different subjects. Although two bending sensor blocks and the data processing circuit block were equally integrated, the misalignment of the sensors with MP joints in the reference device with a fixed design caused malfunction of the device: the signal acquisition accuracy was low and the distinction between different flexion degrees was ambiguous (Figure 4d). On the other hand, the monitoring of the signals was much improved due to the tailored alignment (Figure 4e). Larger capacitance changes were shown at each flexion degree. The measured signals also show remarkable differences between the flexion degrees. This indicates that the sensor blocks are well‐aligned and conformally attached on the MP joints, so that they track the flexion of the joints accurately. We observed similar results for all five subjects (Figure S12, Supporting Information); all subjects performed the flexion tests with the identical sample device fabricated with fixed design. We chose the sensors used in tailored devices with similar initial capacitances to those used in the fixed design. We set the accuracy as the ratio of the capacitance change at 0° to at 90°. The accuracy of the tailored devices was improved by 50% compared to the fixed‐designed devices. (Figure 4f).

In conclusion, we have developed a modular SHE strategy that assembles fundamental electronic blocks on human skins to implement the tailored soft wearable devices. Three types of fundamental building blocks (sensor, interconnect, circuit blocks) were developed to achieve arbitrary system functions regarding data acquisition, data transmission, and data processing. We showed that the engineered strain‐isolation design of each SMEB and the RT bonding technique allowed for rapid and stable tailoring of soft, fully integrated wearable systems. The demonstration we presented here was the first prototype of a system‐level wearable device that was tailored to the form factors of individual bodies. The tailored SHE system, which has been challenging with the existing approaches, provided the functionalities of not only detecting human motion but also displaying direct feedback. We believe that our strategy would open a path forward for the wearable devices that accurately obtain vital signs as well as human activity signals even when worn by people of any age or gender.

## Conflict of Interest

The authors declare no conflict of interest.

## Supporting information

Supplementary

Supplementary

Supplementary

Supplementary
