# Supplementary material for: Soft Modular Electronic Blocks (SMEBs): A Strategy for Tailored Wearable Health‐Monitoring Systems
Source: Adv Sci (Weinh). 2018 Dec 20;6(5):1801682. doi: 10.1002/advs.201801682 (PMC6402283; doi:10.1002/advs.201801682)
Supplement: Supplementary file 1 — Supplementary [file ADVS-6-1801682-s002.pdf]

Copyright WILEY-VCH Verlag GmbH & Co. KGaA, 69469 Weinheim, Germany, 2018.

ADVANCED SCIENCE
Open Access

Supporting Information

for *Adv. Sci.,* DOI: 10.1002/advs.201801682

Soft Modular Electronic Blocks (SMEBs): A Strategy for Tailored Wearable Health-Monitoring Systems

Jaeyoung Yoon, Yunsik Joo, Eunho Oh, Byeongmoon Lee, Daesik Kim, Seunghwan Lee, Taehoon Kim, Junghwan Byun, and Yongtaek Hong**

Copyright WILEY-VCH Verlag GmbH & Co. KGaA, 69469 Weinheim, Germany, 2016.

Supporting Information

On-skin Assembly of Modular Stretchable Hybrid Electronics for Tailored Wearable Healthcare Systems

Jaeyoung Yoon, Yunsik Joo, Eunho Oh, Byeongmoon Lee, Daesik Kim, Seunghwan Lee, Taehoon Kim, Junghwan Byun*, and Yongtaek Hong*

J. Yoon, Y. Joo, E. Oh, B. Lee, D. Kim, S. Lee, Dr. T. Kim, Prof. Y. Hong
Department of Electrical and Computer Engineering
Inter University Semiconductor Research Center (ISRC)
Seoul National University
Seoul 08826, Republic of Korea
E-mail: yongtaek@snu.ac.kr

Dr. J. Byun
Department of Mechanical and Aerospace Engineering
Institute of Advanced Machines and Design (IAMD)
Soft Robotics Research Center (SRRC)
Seoul National University
Seoul 08826, Republic of Korea
E-mail: yongsamarine@snu.ac.kr

Keywords: wearable healthcare devices, stretchable hybrid electronics, on-skin assembly, modular blocks, stretchable platform

Experimental Section

Measurement of hand dimensions: All subjects—50 adult males in their 20s and 30s—were asked to place their hands on a flat and hard surface with fingers extended, and put together their four fingers except thumb. All measurement was executed by one observer to avoid inter-observer bias. Three parameters of hand dimension were measured using vernier calipers as follows[1,2]: (i) Breadth of hand: the distance between the radial side of second MP joint and the ulnar side of fifth MP joint. The maximum distance was measured through a marked line across the four head of metacarpal. (ii) Interval between MP joints: the distance between second and third MP joints. When the MP joints were bent to the maximum angles, the middle points of the MP joints were marked by an observer that are on the front view of dorsum of the hands. Then, the distance was measured with their MP joints extended. (iii) Length of middle finger: the distance between the center of third MP joint and the fingertip of middle finger.

Fabrication of circuit blocks: For preparation of the circuit blocks, PEN flim (thickness =50 μm, Q65H, Teijin DuPont Films) was cut into the proper size and UV/O_3-treated (power = 28 mW cm^{-2}) for 10 minutes for the (3-Aminopropyl)triethoxysilane treatment (ATPES, Sigma-Aldrich). After UV/O_3-treatment, APTES solution was drop casted on the PEN film. After 5 mins in ambient air, the PEN film was rinsed with deionized (DI) water. To remove the remaining APTES solution or DI water, we blew the PEN with N_2 spray gun. After the same process was conducted on the opposite side of the PEN, we obtained APTES-functionalized PEN.

After UV/O_3 treatment for 5 mins on the top side of PEN film, silver electrodes and pads were formed on it using a piezoelectric inkjet printer (DMP-2831, Dimatix Corp.) and annealed at 125°C for 30 mins. Then, silver epoxy is printed at exact positions on pads via pneumatic dispenser (SHOTmini 200Sx, Musashi Engineering, Inc.) or manually and pure

epoxy was also printed between the pads not only to prevent electrical short between silver epoxy but also to bond IC chips to the PEN film robustly.[3-5] After annealing at 185℃ for 30 mins, the bottom side of PEN film and strain-relief PDMS mixed at 10:1 ratio (Sylgard 184, Dow Corning)—All PDMS utilized in this work were annealed at 100℃ for 2 hours—was treated by oxygen plasma (CUTE-1MP, Femto Science) for 60 seconds at a 60W and was bonded to strain relief PDMS to obtain the circuit blocks successfully.

Fabrication of bending sensor blocks: The capacitive-type bending sensor has a similar structure with our previous bending sensor.[6] Bottom plane was fabricated using inkjet printing on the APTES-functionalized PEN film. The ANP ink was printed with dimension of 2×16mm. Dielectric PDMS layer (10:1) was spin-coated on glass at 1000rpm for 60sec, followed by curing on a hotplate. Top plane was flat-structured AgNWs-embedded PDMS, and the fabrication process was conducted with almost the same process of fabricating interconnects blocks. Instead of pre-stretched mold PDMS, a flat glass was used as a mold. The AgNWs of top electrodes were spray-coated with width of 2mm. After preparation of three layers, bottom plane and dielectric PDMS layer were activated through O_2 plasma treatment and put together to form siloxane bond; surrounding region of contact pad of the bottom electrode was not activated due to PDMS masking during O_2 plasma treatment. After exposed to O_2 plasma, top plane was bonded to dielectric PDMS on bottom plane.

Fabrication of interconnect blocks: For preparation of interconnect blocks, mold PDMS (at a ratio of 10:1) was stretched up to 50% uniaxially and UV/O_3 treatment was conducted 45mins. We spray-coated AgNWs solution (YURUI chemical, AgNW length 5-10μm) on the mold dried the AgNWs film at 50℃ for 30 minutes. After releasing the mold PDMS to its initial state (zero strain), the liquid PDMS mixture (10:1 ratio of weight) was casted onto the AgNWs film, followed by curing at 100℃ for 2 hours. Because the liquid PDMS can

penetrate into the void of AgNWs film, the AgNWs film can be embedded into the PDMS after curing process. After peeled off and cut into moderate size, the AgNWs-embedded PDMS with corrugated microstructure—interconnect blocks—were obtained.

180° peel tests: The tests are performed using Instron universal testing machine (Instron-5543, Instron Inc.). The upper layers—APTES-functionalized PEN and 200μm thick 10:1 PDMS—were prepared with dimension of 6×0.5cm. The lower layers—3mm thick 10:1 PDMS and 25:1 PDMS—were prepared with dimension of 6×1cm. Half of the lower layers were masked before applied to oxygen plasma for 1 minute at a power of 60W. Then, the activated surfaces were sandwiched together to form siloxane bonding. After the preparations, the samples were loaded on the 180° peeling setup and the forces were detected with a 50N load cell with a constant peeling speed of 20mm/min. We normalized the averaged the forces by the width of the top substrates to obtain peeling force.

Surface strain mapping based on DIC method: Upon the entire area of not only modular blocks and substrate PDMS, lacquer was sprayed to form randomly distributed speckle patterns. We captured a series of images of interesting area with optical microscope or digital camera while we elongated the samples up to the desired strain level; We used a digital camera for the large area of samples. We evaluate the relative movements of the speckle pattern of each image based on DIC (VIC-2D; Correlated Solutions) method for the given tensile strain. All strain distribution mappings were mapped with maximum principal strain.

Electrical measurement of modular blocks: For measurement of electrical properties of circuit blocks, a SMD MCU, CDC and passive elements (see Table S1 in Supporting Information for detailed chip information) were bonded onto inkjet-printed silver pads via silver epoxy on the APTES-functionalized PEN (25×25mm) of a circuit block. The circuit block was bonded to a

300-μm-thick substrate PDMS and the substrate was slowly elongated up to 30% on the automatic stretching equipment. The voltage at SDA and SCL node were measured by using an oscilloscope (TEKTRONIX DPO4104). For cyclic measurement, the repetitive stretching and releasing test (up to strain of 30%) was conducted at a stretching speed of 300mm min^{-1}. For measurement of characteristics of bending sensor block, the bending sensor block was attached onto automatic bending equipment. The capacitance of the sensor was measured at 1MHz with a 1V a.c. signal by using Agilent 4980a LCR meter during bending deformations. For cyclic measurement, the repetitive bending test (up to bending radius of 6.5mm) was conducted at a bending speed of 10mm min^{-1}. For measurement of electrical properties of interconnect blocks, the substrate PDMS, which had two circuit blocks connected by an interconnect block (2×20mm), was elongated up to 50% strain. While the substrate was elongated, electrical resistance of the interconnect block was continuously measure by Keithley 2420 and Labview program.

MP joint flexion monitoring system: A signal-process circuit block, two LED gauge blocks, and two bending sensor blocks, and interconnect blocks were prepared, and their circuit schematic is shown in Figure S6 in Supporting Information. After O_2 plasma treatment onto not only all modular blocks but also substrate PDMS, substrate PDMS was attached on dorsum of hand using silicone gel adhesive (PS-2055, Polymer science, Inc.). The circuit and sensor blocks were arranged on a proper position of hand and physically contacted with substrate PDMS. Interconnect blocks were cut into proper length matched to the distance between contact pads of circuit or sensor blocks and bonded onto not only substrate PDMS but also each block. After that, the contact pad and interconnect blocks were electrically connected by silver paste.

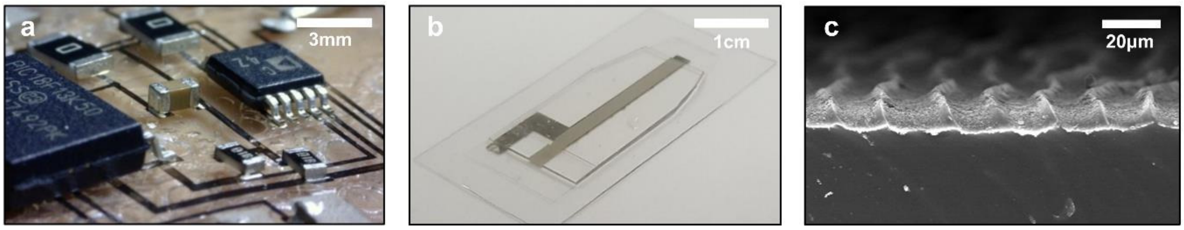

Figure S1. Soft modular electronic blocks (SMEBs). a) Optical image of a circuit block. b) Photograph of a bending sensor block. c) SEM image of a interconnect block

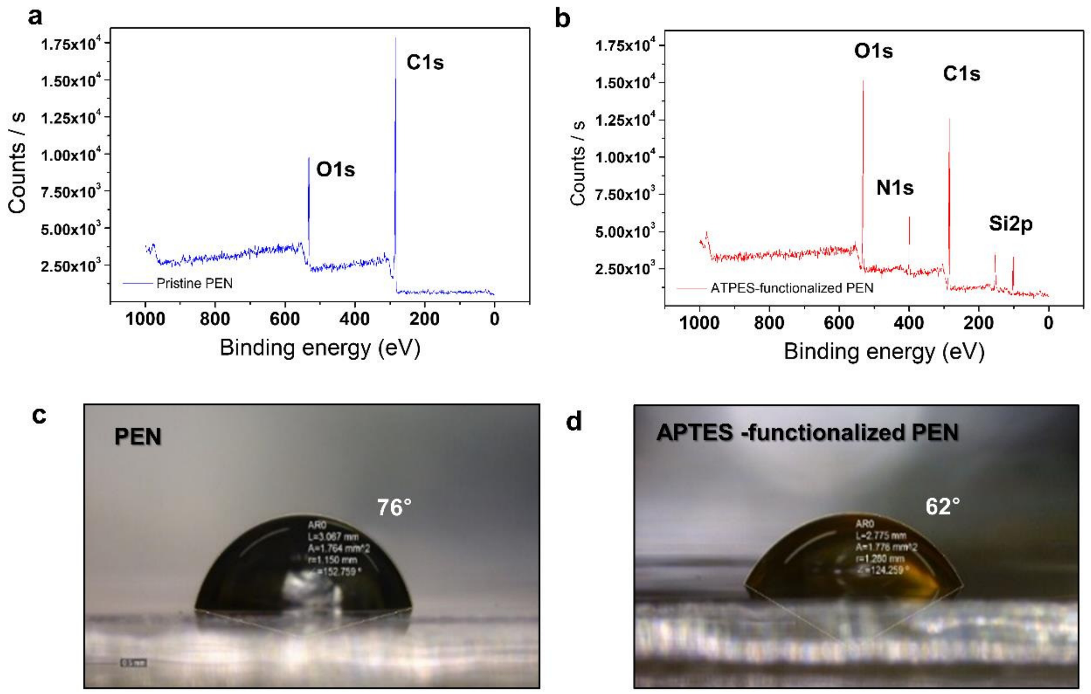

Figure S2. Surface modification of a PEN island with APTES treatment. XPS spectra of (a) pristine PEN film surface and (b) APTES-functionalized PEN film surface. XPS analyses were performed on a SIGMA PROBE (ThermoVG, UK). Cross-sectional images of water droplets on (c) a pristine PEN film surface and (d) a APTES-functionalized PEN film surface.

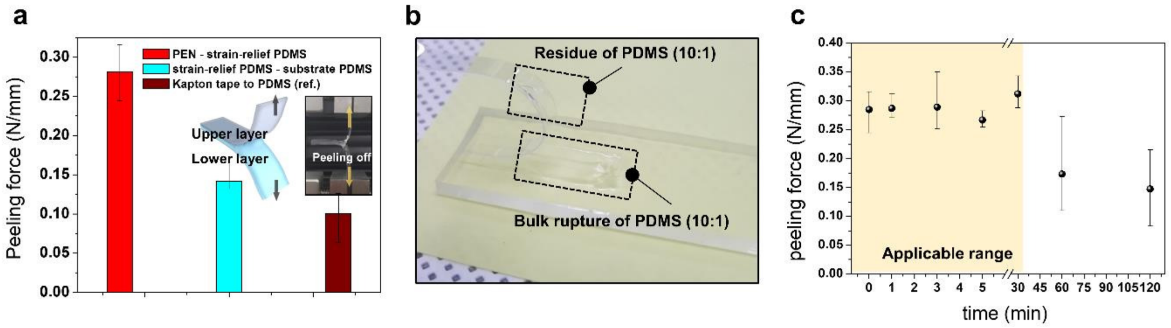

Figure S3. a) Bonding strength of the layers in our system. (Insets images: illustration and photograph of 180° peel-off test). b) Photograph of a PEN-bonded PDMS sample after peeling-off test. c) Peeling force versus lapse of time before the physical attach between plasma-activated PEN and PDMS. We set the applicable time range as 30 minutes.

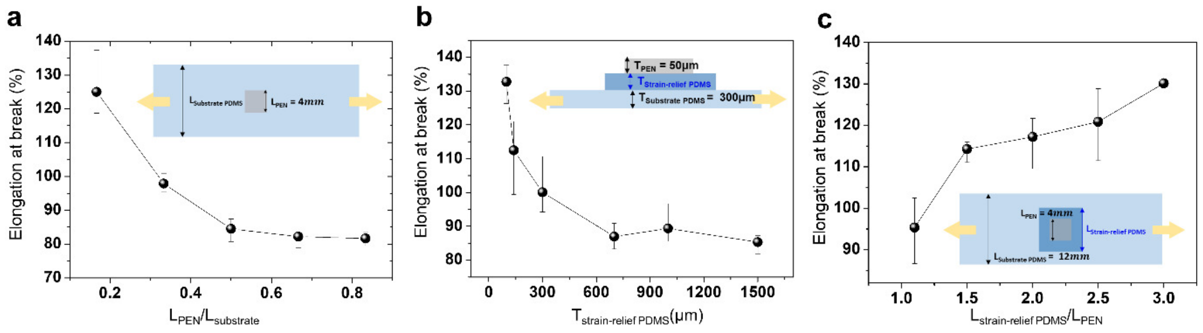

Figure S4. Tensile stretch test using an Instron universal testing machine. a) A correlation graph between elongation at break and the ratio of width of PEN to PDMS (in this case, the experimental samples had no strain-relief PDMS). Correlation graphs between elongation at break and the ratio of (b) thickness and (c) width of strain-relief PDMS to those of PEN.

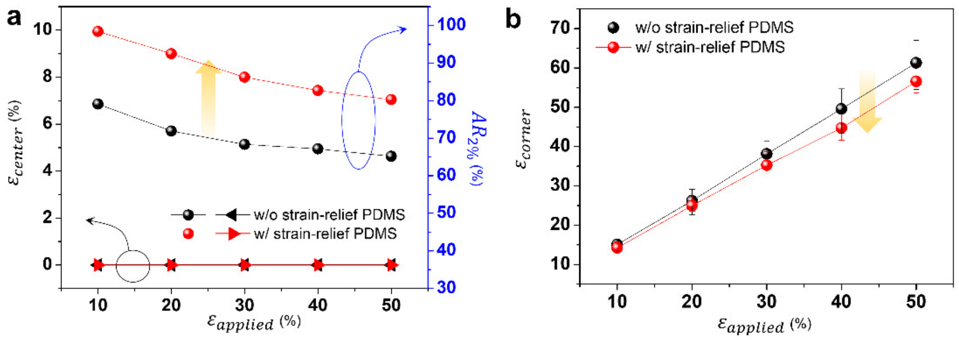

Figure S5. Mechanical stability of assembled SMEBs. The effect of a strain-relief PDMS layer on the stability of (a) the inside and (b) the boundary of the islands.

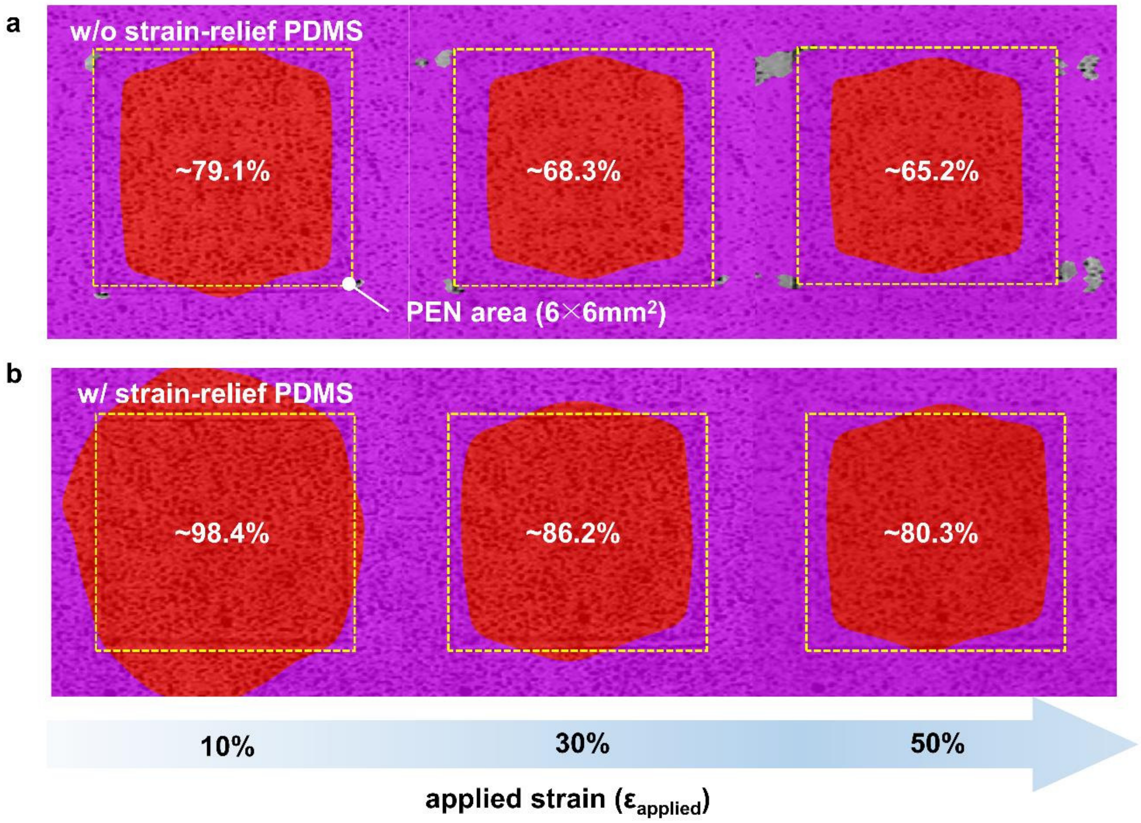

Figure S6. $AR_{2\%}$ of the structure (a) without and (b) with the strain-relief PDMS layer as a function of the applied uniaxial strain ($\varepsilon_{applied}$= 0~50%). Surface strain mapping is evaluated from a DIC method, and the $AR_{2\%}$ values are calculated from image processing. Areas within a strain level of 2% are visualized as red domains.

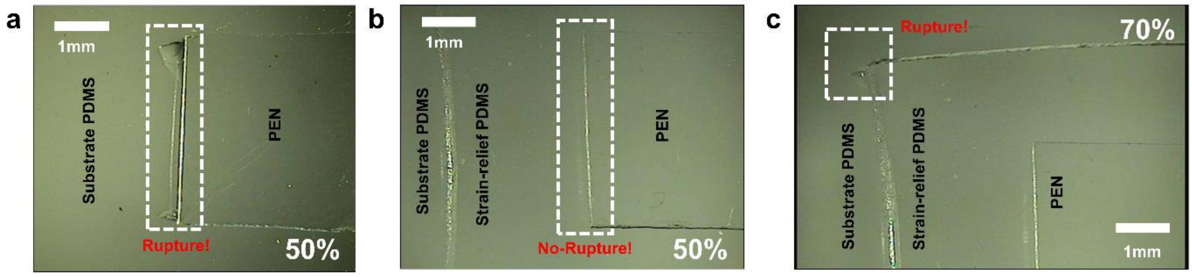

Figure S7. Optical images of the structures without and with the strain-relief PDMS layer. a) The onset of the PDMS rupture at the corner of PEN under uniaxial strain of 50% without the strain-relief layer. b) No PDMS rupture at the corner of the PEN island under 50% uniaxial strain and (c) the onset of the PDMS rupture at the corner of the strain-relief layer under 70% uniaxial strain.

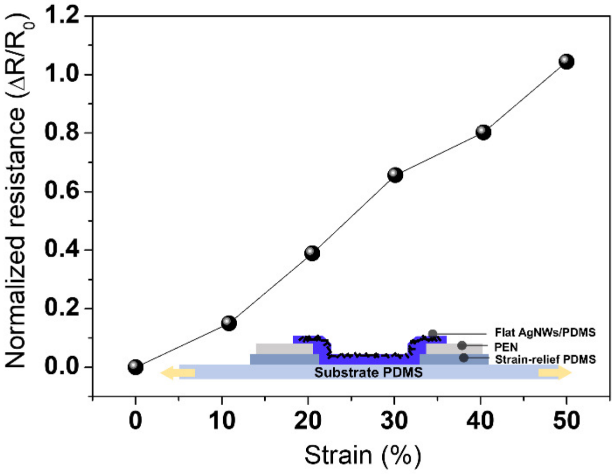

Figure S8. Resistance change of the flat-structured interconnect block as a function of strain (0-50%).

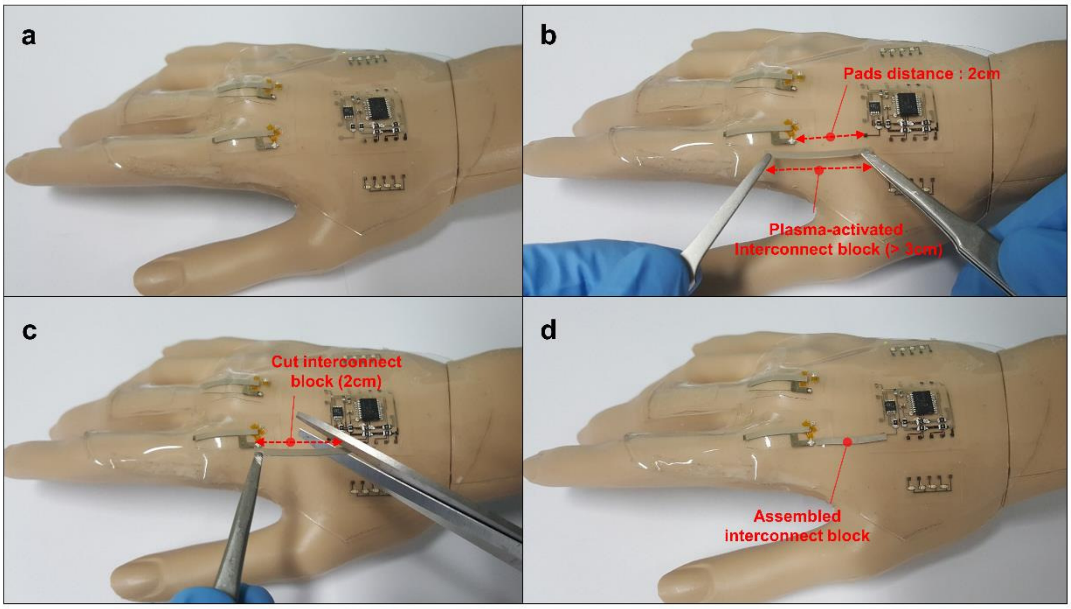

Figure S9. Tailoring of interconnect blocks according to the layout of SMEBs. a) Arbitrary layouts of the circuit and sensor blocks. b) Determination of length of an interconnect block by comparing the distance between the two pads. c) Cutting the interconnect block into the suitable length by scissors. d) Assembling the interconnect blocks.

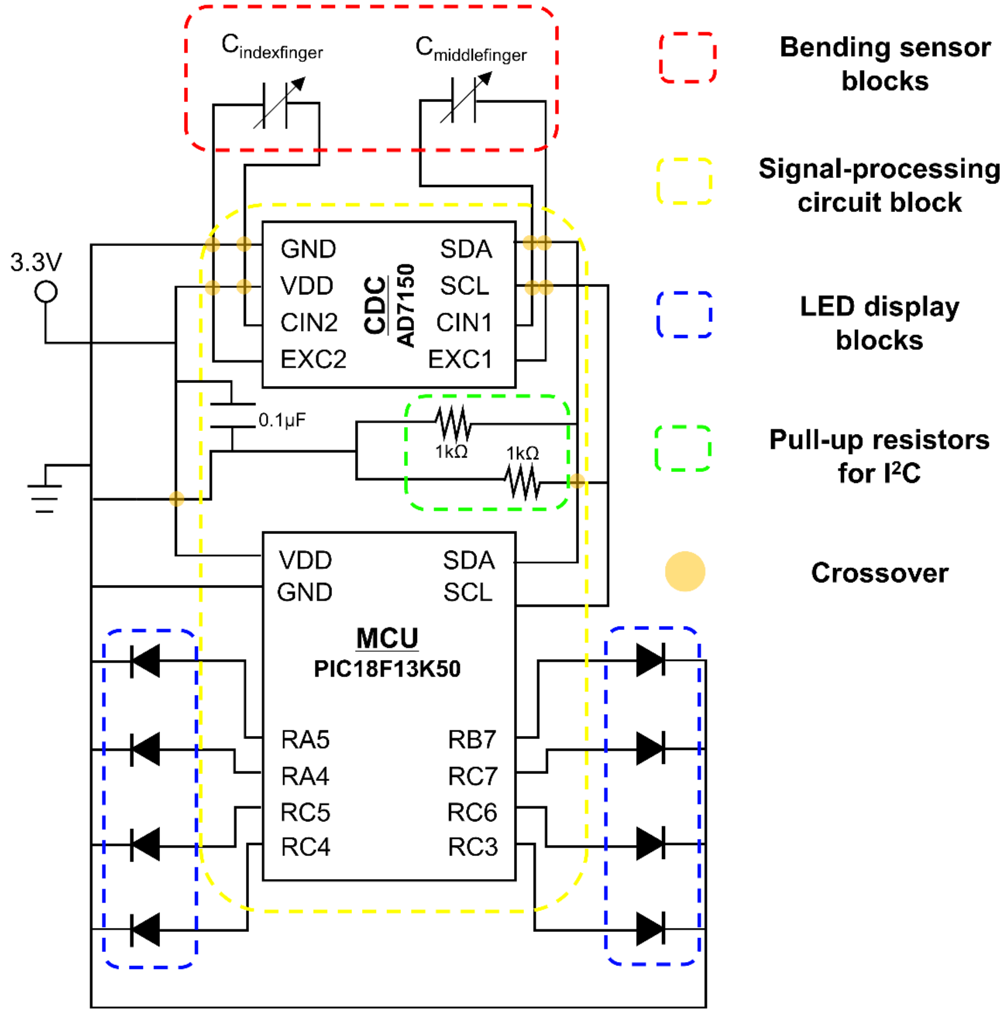

Figure S10. Circuit diagram of a MP joints flexion monitoring system. A micro-controller unit (MCU) was programmed to communicate with a capacitance-to-digital converter (CDC) through I^2C. The bending sensor data are updated every 20 ms and comprared with the calibrated data. After comparison, the MCU turns LED chips on and off according to the results.

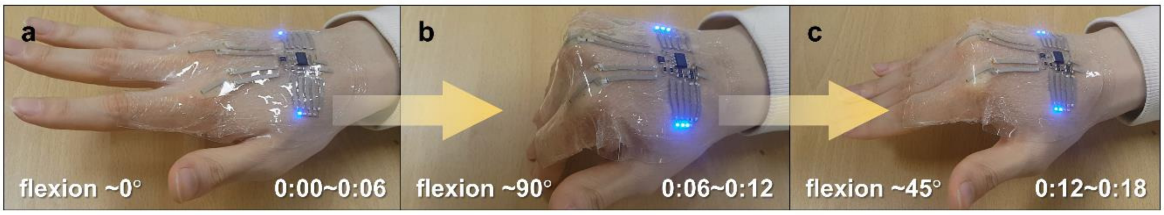

Figure S11. Calibration process of a MP joints flexions monitoring device. Calibration begins when the external power is applied. Right after that, the LED display block blinks for (a) one, (b) three, and (c) two LEDs for 6 seconds each, indicating that calibration is in progress. We asked the subject to bend the MP joints at 0, 90, and 45° at each step (a-c).

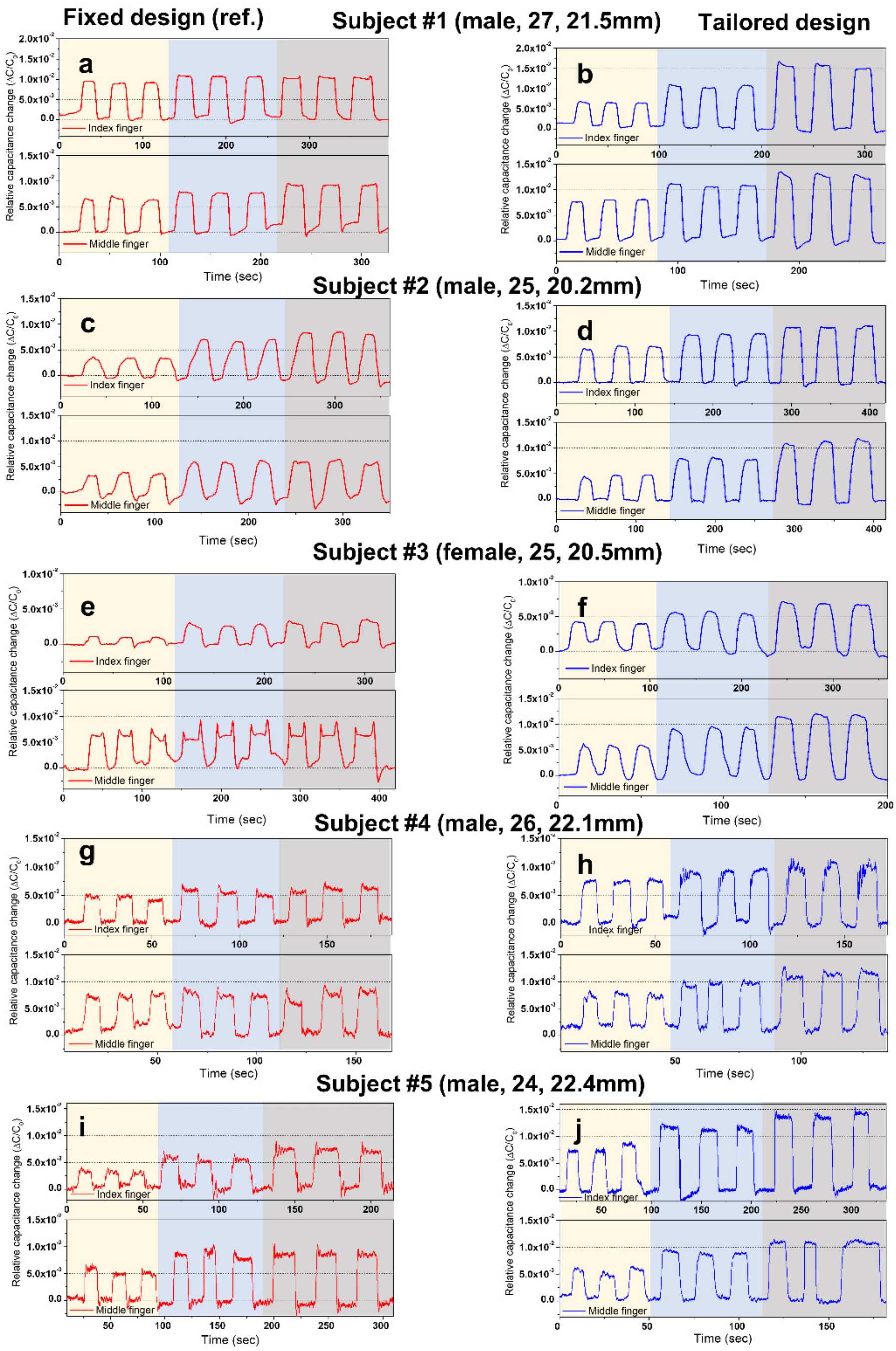

Figure S12. The sensor signals that monitor the hand flexion to 30, 60, 90° of the wearable device with (a,c,e,g, and i) a fixed and (b,d,f,h, and j) a tailored design for five different subjects. The personal data and form factors of the subjects are shown in the following formats: (Sex, age, distance between 2nd and 3rd MP joints)

Product name	Company	Description	Dimension(mm^3)
PIC18F13K50-I/SS	Microchip Technology	IC MCU 8BIT 8KB FLASH 20SSOP	7.2×7.8×2
AD7150BRMZ	Analog Devices Inc.	IC CAP CONV 2CH ULT LP 10MSOP	3×4.9×1.1
LTST-C193TBKT-5A	Lite-On Inc.	LED BLUE CLEAR 0603 SMD	1.6×0.8×0.35
RC0805JR-070RL	Yageo	RES SMD 0 OHM JUMPER 1/8W 0805	2×1.25×0.5
CR0603-FX-1001ELF	Bourns Inc.	RES SMD 1K OHM 1% 1/10W 0603	1.6×0.8×0.45
0603YC104JAT2A	AVX Corporation	CAP CER 0.1UF 16V X7R 0603	1.6×0.81×0.9

Table S1. Chip information used in this work

References

[1] T. Kanchan, P. Rastogi, J. Forensic Sci. **2009**, 54, 3.

[2] K. Krishan, T. Kanchan, A. Sharma, J. Forensic Sci. **2011**, 56, 2.

[3] J. Byun, B. Lee, E. Oh, H. Kim, S. Kim, S. Lee, Y. Hong, Sci. Rep. **2017**, 7, 45328.

[4] J. Byun, E. Oh, B. Lee, S. Kim, S. Lee, Y. Hong, Adv. Funct. Mater. **2017**, 27, 1701912.

[5] J. Byun, Y. Lee, J. Yoon, B. Lee, E. Oh, S. Chung, T. Lee, K.-J. Cho, J. Kim, Y. Hong, Sci. Robotics **2018**, 3, eaas9020.

[6] Y. Joo, J. Yoon, J. Ha, T. Kim, S. Lee, B. Lee, C. Pang, Y. Hong, Adv. Electron. Mater. **2017**, 3, 1600517.
